# Supplementary material for: Association between antenatal diagnosis of late fetal growth restriction and educational outcomes in mid-childhood: A UK prospective cohort study with long-term data linkage study
Source: PLoS Med. 2023 Apr 24;20(4):e1004225. doi: 10.1371/journal.pmed.1004225 (PMC10166482; doi:10.1371/journal.pmed.1004225)
Supplement: S1 Appendix — Analysis plan for assessing whether prenatal diagnosis of late fetal growth restriction is predictive of later educational achievement. (DOCX) [file pmed.1004225.s001.docx]

**S1 Appendix. Data analysis plan**

**Analysis plan for assessing whether prenatal diagnosis of late fetal growth restriction is predictive of later educational achievement**

1. **Introduction**

Early delivery of small for gestational age (SGA) infants is associated with poorer educational achievement in childhood[1]. SGA infants can broadly be sub-classified into “healthy small” and those fetal growth restriction (FGR) and it is unclear whether associations with SGA are wholly mediated through its association with FGR. Moreover, it is currently unclear whether the association is intrinsic, i.e. SGA and/or FGR predisposes to poorer educational outcome, or iatrogenic, i.e. earlier delivery predisposes to poorer educational outcome. Retrospective analyses of cases of FGR are limited by the effect of clinician knowledge of the results of ultrasonic assessment and consequent intervention. The Pregnancy Outcome Prediction Study (POPS) performed serial blinded assessment of fetal growth and measurement of both biochemical and ultrasonic indicators of fetal growth restriction in >4000 women having first pregnancies. The dataset is a unique resource for determining the natural history of diagnosis of FGR and it has now been linked to educational data obtained from the National Pupil Database (NPD) in the UK Department for Education.

1. **Research questions**
2. Do SGA fetuses with ultrasonic and/or biochemical indicators of FGR detected at 36 weeks of gestation have an increased risk of poor educational attainment following delivery at term?
3. Do SGA fetuses without ultrasonic and biochemical indicators of term FGR detected at 36 weeks of gestation have an increased risk of poor educational attainment following delivery at term?
4. Do appropriate for gestational age (AGA) fetuses with ultrasonic and biochemical indicators of term FGR detected at 36 weeks of gestation have an increased risk of poor educational attainment following delivery at term?
5. **Defining datasets**
6. Pregnancy Outcome Prediction Study (POPS) dataset, Department of Obstetrics & Gynaecology, University of Cambridge

POPS was a prospective cohort study of 4512 nulliparous women attending antenatal visits at the Rosie Maternity Hospital, Cambridge, England between January 2008-July 2012[2]

There were 4 POPS study visits during pregnancy[2]: 1) at 12 weeks consisted of taking informed consent and phlebotomy, 2) at 20 weeks consisted of fetal biometry, phlebotomy, uterine and umbilical Doppler, 3) at 28 weeks consisted of fetal biometry, phlebotomy, uterine and umbilical Doppler, and 4) at 36 weeks consisted of fetal biometry, phlebotomy, uterine and umbilical Doppler.

Data to use: pregnancy/antenatal factors (fetal growth parameters from antenatal ultrasounds, other biomarkers), maternal data (age, morbidities, parity, occupation, partner status, etc), perinatal information (gestational age, infant sex, birthweight, etc), other baseline demographics (including socioeconomic parameters).

4164 participants who had live born singleton infants were eligible for inclusion. List cleaning reduced this to 3722 mother/baby pairs who were confirmed alive and traceable at study commencement. 45 pairs opted out, therefore linkage was attempted for 3677 pairs.

1. National Pupil Database (NPD) datasets, Department for Education

Data to use: early years, phonics, key stage 1

1. NHS digital data datasets

Data to use: hospital episode statistics (HES) at outpatient clinic, inpatient ward, or A&E unit to exclude children with significant morbidities that can interfere with their academic performance but are unlikely to be causally linked to FGR, i.e. any child with evidence of these diseases: 1) major congenital anomaly, genetic, or chromosomal difference; 2) neurological or cerebrovascular disease; 3) childhood malignancy; 4) inborn errors of metabolism or immunodeficiency; 5) congenital hearing impairment or visual loss; early onset of severe organ dysfunction; 6) dependency on medical machines, functional implants, artificial feeding or breathing.

Children without HES data during the time frame (1 year) are assumed to not have any major medical morbidities. There may be a small number of children who were being managed entirely via the private health care system or not have required any hospital management at all over the course of a year, however given the medical complexity of the exclusion criteria, this is unlikely and would only apply to a very small number of children.

All data sets are identified only by anonymised study ID

1. **Defining exposures**

How does educational attainment at age 5-7 years differ between the following four groups?

1. Ultrasonic SGA with indicators of fetal growth restriction
2. Ultrasonic SGA without indicators of fetal growth restriction
3. Ultrasonic AGA without indicators of fetal growth restriction (referent)
4. Ultrasonic AGA with indicators of fetal growth restriction

**SGA** = small-for-gestational age, defined as EFW <10^th^ percentile based on relevant growth reference, adjusted for sex and GA (as per Sovio et al, 2015[3]). Analyses will be repeated for EFW <20^th^ as this will include a larger proportion of all infants with SGA birth weight.

**AGA** = appropriate-for-gestational age, defined as EFW >10^th^ and <90^th^ percentiles. When defining SGA as <20^th^, AGA will be defined as >20^th^ and <90^th^.

All infants included in the analyses were born at term (gestational age > completed 37 weeks).

**Ultrasonic evidence of fetal growth restriction is defined as one or more of the following:**

1. lowest decile of abdominal circumference (AC) velocity, if the change in the AC z-score between 20 and 36 weeks <10^th^ percentile
2. EFW <3^rd^ percentile
3. High resistance uterine Doppler flow velocimetry (mean uterine artery pulsatility index at 20 weeks >90^th^ percentile
4. High resistance umbilical artery Doppler flow velocimetry (umbilical artery pulsatility index z-scores at 36 weeks >90^th^ percentile

The thresholds employed in the current analysis have been previously defined (Sovio et al, 2015[3]).

**Biochemical evidence of fetal growth restriction is defined as one or more of the following:**

1. Maternal serum level of PAPP-A <0.4 MoM in early pregnancy/at 12 weeks of gestational age (assay and quantification as defined by Hughes et al 2019)[4]
2. Maternal serum sFlt-1:PlGF ratio >38 at 36 weeks of gestational age (assay and quantification as defined by Sovio et al, 2017)[5]
3. Maternal serum level of AFP >2.0 MoM in the second trimester/20 weeks of gestational age (assay and quantification as defined by Hughes et al 2019)[4]
4. **Defining outcomes: educational attainment at age 5-7 years**

**Early years foundation stage (EYFS)**, representing educational attainment at the age of 5 with 7 domains of assessment: 3 prime areas consisting of communication and language (CL), physical development (PHY), personal-social-emotional development (PSE), and 4 specific areas consisting of literacy (LIT), mathematics (MAT), understanding the world (UTW), expressive arts and design (EXP). There are in total 17 early learning goals (ELG) of those 7 domains of EYFS. Children were assessed at the end of reception year to be either at an ‘emerging’ (=1), ‘expected’ (=2), or ‘exceeding’ (=3) level of development in the 17 ELG, making 17 as minimum and 51 as maximum scores, respectively. Good level of development (GLD) is achieved if children have *at least* the expected level for the ELG in CL, PHY, PSE, LIT, and MAT.

**Phonics** assessment, representing educational attainment at the age of 6 (end of year 1), is measured by using 40-word test with national threshold for pass is >32. Children who did not pass would repeat this assessment at the end of year 2.

**Key stage 1** (KS1), representing educational attainment at the age of 7 (end of year 2), consisting of reading, writing, mathematics, and science tests. Corresponding teachers would judge the test results compared to standard: ‘below’, ‘at’, ‘above expected’.

**Table 1 Type of each outcome variable analysis plan**

| **Dependent variables/Outcomes:**  **School outcomes at age 5-7 years** | |
| --- | --- |
| **Variable** | **Type** |
| Early years | Binary categories: achieved GLD v. non-GLD |
| Phonics assessment | Binary categories: first-time passed v. failed |
|  |  |
| Key stage 1 | Binary categories: below v. at/above, per domain being assessed: reading, writing, mathematics, science |

The analyses will be repeated using the full range of values for each of the above assessments, i.e. In the case of early years and phonics, this will employ multiple linear regression following transformation of the dependent variable to a normal distribution, if required. In the case of KS1, this will employ ordinal logistic regression for each of the domains.

1. **Defining maternal occupation data handling**

Maternal occupations were coded into 9 groups based on ISCO-08 - ONS SOC Hierarchy 2010 (onsdigital.github.io):

1 = Managers, Directors, and Senior Officials; 2 = Professional occupations; 3 = Associate Professional and Technical Occupations; 4 = Administrative and Secretarial Occupations, 5 = Skilled Trades Occupations; 6 = Caring, Leisure, and Other Service Occupations; 7 = Sales and Customer Service Occupations; 8 = Process, Plant, and Machine Operatives; 9 = Elementary occupations

and 2 categories were additionally derived:

0 = refers to all occupations with no stable regular income, including unemployed, housewife or homemaker, student, voluntary work; and 10 = self-employed without any other information

These 10 occupation groups were then modified into 4 categories:

M = managerial occupations or group 1; P = professional occupations or group 2; A = Associate and administrative occupations or group 3 and 4; E = occupations that require elementary-level educations or group 5, 6, 7, 8, and 9; U = refers to un-, freelance, or self-employment (excluding business owner)

1. **Defining covariates**

**Table 2 List of covariates used in the analyses**

|  | **Variable** | **Type** | **Notes** |
| --- | --- | --- | --- |
| **Maternal factors** | Age | Numeric (continuous) | Derived from date of POPS of recruitment – date of birth (in years) |
|  | Maternal BMI | Numeric (continuous) | Derived from maternal weight at recruitment divided by the square of measured height (in kg/m^2^) |
|  | Ethnicity | Factor (binary categories) | White (Caucasian) v. non-white |
|  | Occupation | Factor | Group 1, 2, 3, 4 (see point 6 – maternal occupation data handling) |
|  | Partner status | Factor (binary categories) | Yes/no |
|  | Smoking history | Factor | “never smoked”, “quit during pregnancy”, “quit before pregnancy”, and “currently smoking during pregnancy” (could further be grouped into binary variables: smoking v. not smoking during pregnancy) |
| **Infant factors** | Gestational age | Numeric (continuous) | In weeks, taking into account remaining days as fraction of corresponding number of weeks, e.g. 37 weeks and 3 days = 37.4 weeks |
|  | Sex | Factor (binary categories) | Male/female |
|  | Birth seasonality | Factor | 1. born between 1st of December at 00:00 to 28th (or 29th during a leap year) of February at 23:59 (representing winter) 2. born between 1st of March at 00:00 to 31st of May at 23:59 (representing spring) 3. born between 1st of June at 00:00 to 31st of August at 23:59 (representing summer) 4. born between 1st of September at 00:00 to 30th of November at 23:59 (representing autumn) |
| **Socioeconomic factors** | Index of multiple deprivation (IMD) | Numeric (continuous) | Based on the English indices of deprivation 2007 (postcode-based) |
|  | School funding type | Factor | Academy (state-funded), community, voluntary |
|  | Year | Factor | Year when the assessment being conducted (all outcomes will be adjusted for year, *except* numerical total EY z-scores) |
| **Childhood physical health** | Relevant childhood comorbidities | Factor (binary categories) | Any evidence of these diseases, recorded from hospital episode statistics (HES)-NHS digital from either A&E attendances, outpatient appointments, or inpatient admissions at NHS hospitals in England: 1) major congenital anomaly, genetic, or chromosomal difference; 2) neurological or cerebrovascular disease; 3) childhood malignancy; 4) inborn errors of metabolism or immunodeficiency; 5) congenital hearing impairment or visual loss; early onset of severe organ dysfunction; 6) dependency on medical machines, functional implants, artificial feeding or breathing |

1. **Defining general analytical plan**

**Figure 1 Pre-analysis flowchart**

Notes:

1. Full outer join/merge will return all rows/participants from each dataset and match up rows where possible
   - We will exclude infants born <37 weeks of gestation to exclude the effect of prematurity
   - Participants without any educational data in the NPD will also be omitted from the analyses.
2. Multiple imputations by chained equations (MICE)[6] will be employed to replace covariates’ missing data values under assumption that the data are missing at random (MAR).

**Figure 2 Strategy of data analyses**

*3 models will be constructed based on NPD academic outcomes: 1) early years, 2) phonics, 3) Key Stage/KS1

**Statistical methodology**

| **Methods** | **Outcomes** |
| --- | --- |
| Descriptive analyses | Descriptive comparisons between groups   - For numerical variables (normal distribution presented in mean +standard deviation; non-normal distribution in median (interquartile range) - For categorical variables (in N and %) |
| Correlation analyses | Correlation coefficients and *p values* (Pearson’s for normal distribution and Spearman’s for non-normal distribution) |
| Linear and non-linear regression models (including generalized additive models) | B coefficients, standard errors, and *p values* |
| Logistic regression models | Odd ratios, confidence intervals, and *p values* |

1. **Sensitivity and additional analyses**

All analyses will be repeated using the threshold of EFW <20^th^ percentile.

We will also explore associations between each of the markers of FGR in combination with EFW <20^th^ to determine whether associations vary with the classifier of FGR.

Analyses will be repeated excluding women who had any clinically indicated ultrasound scan performed >35 weeks of gestational age. The aim is to remove any potential for effects of clinical intervention caused by knowledge of fetal growth status by the attending clinicians due to a clinically-indicated ultrasound examination.

Sensitivity analyses will also be performed by including those with severe childhood morbidities (defined as above); both with and without adjusting for childhood morbidities in the analysis

1. **Quality control**

Regular weekly/fortnightly meetings will be conducted between LO and CA to discuss ongoing data analyses. Data will be discussed with all other POPStar study team members once ready and being released from the SRS.

**References**

1. Selvaratnam RJ, Wallace EM, Wolfe R, Anderson PJ, Davey MA. Association between Iatrogenic Delivery for Suspected Fetal Growth Restriction and Childhood School Outcomes. JAMA. 2021;326(2):145–53.

2. Gaccioli F, Lager S, Sovio U, Charnock-Jones DS, Smith GCS. The pregnancy outcome prediction (POP) study: Investigating the relationship between serial prenatal ultrasonography, biomarkers, placental phenotype and adverse pregnancy outcomes. Placenta. 2017;59(Suppl 1):S17–25.

3. Sovio U, White IR, Dacey A, Pasupathy D, Smith GCS. Screening for fetal growth restriction with universal third trimester ultrasonography in nulliparous women in the Pregnancy Outcome Prediction (POP) study: A prospective cohort study. Lancet. 2015;386(10008):2089–97.

4. Hughes AE, Sovio U, Gaccioli F, Cook E, Charnock-Jones DS, Smith GCS. The association between first trimester AFP to PAPP-A ratio and placentally-related adverse pregnancy outcome. Placenta. 2019;81(April):25–31.

5. Sovio U, Gaccioli F, Cook E, Hund M, Stephen Charnock-Jones D, Smith GCS. Prediction of Preeclampsia Using the Soluble fms-Like Tyrosine Kinase 1 to Placental Growth Factor Ratio: A Prospective Cohort Study of Unselected Nulliparous Women. Hypertension. 2017;69(4):731–8.

6. Rubin D. Multiple imputation for nonresponse in surveys. Hoboken, New Jersey: John Wiley & Sons; 2008.
