# Supplementary material for: Association between antenatal diagnosis of late fetal growth restriction and educational outcomes in mid-childhood: A UK prospective cohort study with long-term data linkage study
Source: PLoS Med. 2023 Apr 24;20(4):e1004225. doi: 10.1371/journal.pmed.1004225 (PMC10166482; doi:10.1371/journal.pmed.1004225)
Supplement: S2 Appendix — (DOCX) [file pmed.1004225.s002.docx]

**S2 Appendix. Assessment of educational attainment aged 5, 6, and 7 years in the UK**

Educational outcomes were obtained from the National Pupil Database, a national record-level data resource curated by the UK Department for Education (DfE). All fully/partially state-funded schools in the England have a mandatory reporting requirement to return individual-level pupil data on an annual basis.

In the UK, children typically start school/reception (also called Year 0 of primary school) in September after their 4^th^ year birthday. They must have started full-time education by the start of the next term following their 5^th^ birthday. Therefore, the vast majority of children will be aged 5, 6, and 7 respectively in the first 3 school years.

Assessment aged 5

At the end of the first school year (Year 0/Reception), educational attainment is assessed in 7 key areas, divided into 17 early learning goals (ELG) (see table below). For each ELG, children are assessed as ‘emerging’ (=1), ‘expected’ (=2), or ‘exceeding’ (=3) level of development. The minimum possible score is therefore 17 and the maximum is 51. Scores are assigned on the basis of ongoing observations by the class teacher.

The standardised binary outcome reported by the Department for Education is ‘Good level of development’ (GLD). This is achieved if children attain the expected level or above for the ELGs in the areas of (i) communication and language, (ii) physical development, (iii) personal, social, and emotional development, (iv) literacy, and (v) mathematics.

| **No** | **Key areas** | **Early learning goals** | **Included as parameters of ‘Good Learning Development’** |
| --- | --- | --- | --- |
| 1 | **Communication and language** | 1. Listening & attention 2. Understanding 3. Speaking | V |
| 2 | **Physical development** | 1. Moving and handling 2. Health and self-care | V |
| 3 | **Personal, social, and emotional development** | 1. Self-confidence and self-awareness 2. Managing feelings and behaviour 3. Making relationships | V |
| 4 | **Literacy** | 1. Reading 2. Writing | V |
| 5 | **Mathematics** | 1. Numbers 2. Shape, space, and measures | V |
| 6 | **Understanding the world** | 1. People and communities 2. The world 3. Technology | X |
| 7 | **Expressive arts, designing, and making** | 1. Exploring and using media and materials 2. Being imaginative | X |

Assessment aged 6

Towards the end of the second school year (Year 1), pupils are assessed using a standardised 40-word test, administered by a trained education professional known to the child. This assessment results in a numerical global score (1-40) for each child, which is then converted into a binary outcome by comparing their performance to a national threshold (set on a yearly basis, usually ~32). Children who do meet the national threshold aged 6 are given the opportunity to repeat this test aged 7, however in our analysis we used only the scores from each child’s first attempt aged 6.

Assessment aged 7

At the end of the third school year (Year 2), children are assessed by a teacher who has had contact with the pupil over a period of time. Four key domains (reading, writing, mathematics, and science) are assessed as either ‘below’, ‘at’, or ‘above’ expected standards. A binary outcome (below *versus* at or above standard) was generated for each KS1 domain.

**Further details of assessment criteria, standardisation of testing, and audit/validation methods can be found in the following documents:**

Department for Education. *National Pupil Database.* https://www.gov.uk/government/collections/national-pupil-database. Published 2018. Accessed November 20, 2021.

Department for Education. *Early years foundation stage profile results: 2018 to 2019*; <https://www.gov.uk/government/statistics/early-years-foundation-stage-profile-results-2018-to-2019>. Published 17 October 2019, Last updated 7 April 2020. Accessed 4 May, 2022.

Department for Education. *National curriculum assessments at key stage 1 and phonics screening checks in England, 2018;* <https://www.gov.uk/government/statistics/phonics-screening-check-and-key-stage-1-assessments-england-2018/national-curriculum-assessments-at-key-stage-1-and-phonics-screening-checks-in-england-2018>. Published 27 September 2018, Updated 25 October 2019. Accessed 4 May 2022.

Office of Qualifications and Examinations Regulation (Ofqual). *National assessments regulation annual report 2019;* <https://www.gov.uk/government/publications/national-assessments-regulation-annual-report-2019>. Published 28 January 2020. Accessed 4 May 2022.

Office of Qualifications and Examinations Regulation (Ofqual). *Regulatory framework for national assessments, National curriculum and early years foundation stage assessments;* https://assets.publishing.service.gov.uk/government/uploads/system/uploads/attachment_data/file/685610/National_assessment_regulatory_framework_-_March_2018.pdf . Published March 2018. Accessed 4 May 2022.
