# Supplementary material for: Association between antenatal diagnosis of late fetal growth restriction and educational outcomes in mid-childhood: A UK prospective cohort study with long-term data linkage study
Source: PLoS Med. 2023 Apr 24;20(4):e1004225. doi: 10.1371/journal.pmed.1004225 (PMC10166482; doi:10.1371/journal.pmed.1004225)
Supplement: S1 Table — These conditions are not linked to intrauterine development but could impact childhood educational performance and therefore were adjusted in primary analyses. This prespecified morbidity list was defined in consultation with a paediatric consultant (HW). A full year of hospital episode statistics (HES) was obtained for each child, as it is highly likely that any child with a significant excludable health condition would have at least one HES-recorded appointment within a year. Children without any HES data recorded during the time frame (1 year) are assumed to not have any of the prespecified morbidities. There may be a small number of children who were being managed entirely via the private healthcare system or not have required any hospital management at all over the course of a year; however, given the medical complexity of the prespecified conditions, this is unlikely and would only apply to a very small number of children. (DOCX) [file pmed.1004225.s004.docx]

**S1 Table. Childhood medical conditions for model adjustment**

| - Major congenital anomaly, genetic, or chromosomal difference |
| --- |
| - Neurological or cerebrovascular disease |
| - Childhood malignancy |
| - Inborn errors of metabolism or immunodeficiency |
| - Congenital hearing impairment or visual loss |
| - Early onset of severe organ dysfunction |
| - Dependency on medical machines, functional implants, artificial feeding/breathing |

These conditions could confound the association between disrupted intrauterine environment and reduced educational performance and therefore were adjusted in primary analyses. This pre-specified morbidity list was defined in consultation with a paediatric consultant (HW). A full year of hospital episode statistics (HES) was obtained for each child, as it is highly likely that any child with a significant excludable health condition would have at least one HES-recorded appointment within a year. Children without any HES data recorded during the time frame (1 year) are assumed to not have any of the pre-specified morbidities. There may be a small number of children who were being managed entirely via the private health care system or not have required any hospital management at all over the course of a year, however given the medical complexity of the pre-specified conditions, this is unlikely and would only apply to a very small number of children.
