# Supplementary material for: Association between antenatal diagnosis of late fetal growth restriction and educational outcomes in mid-childhood: A UK prospective cohort study with long-term data linkage study
Source: PLoS Med. 2023 Apr 24;20(4):e1004225. doi: 10.1371/journal.pmed.1004225 (PMC10166482; doi:10.1371/journal.pmed.1004225)
Supplement: S2 Table — Abbreviations: AC, abdominal circumference; AFP, alpha-feto protein; EFW, estimated fetal weight; PAPP-A, pregnancy-associated plasma protein-A; sFlt1:PlGF, soluble fms-like tyrosine kinase 1:placental growth factor ratio; UMB-PI, umbilical artery pulsatility index; UT-PI, uterine artery pulsatility index. (DOCX) [file pmed.1004225.s005.docx]

**S2 Table. Ultrasonic biometry and markers of placental dysfunction of all POPS participants and the analytic sample**

|  | **Analytic sample**  **(N=2754)** | **Excluded participants**  **(N=1410)** | **All POPS participants (N=4164)** |
| --- | --- | --- | --- |
| EFW centile, mean (SD) | 38·56 (25·0) | 47·16 (31·53) | 41·05 (27·32) |
| Frequency of EFW <10^th^ centile, No. (%) | 376 (13·7) | 144 (10·2) | 542 (13) |
| Frequency of EFW <3^rd^ centile, No. (%) | 66 (2·4) | 41 (2·9) | 132 (3·2) |
| Missing, No. (%) | <10 | 297 (21·1) | 297 (7·1) |
| AC growth between 20-36 wk-z score, mean (SD) | -0·04 (0·99) | 0·1 (1·02) | 0·001 (1·0) |
| Frequency of low AC growth between 20-36 wk, No. (%) | 304 (11) | 94 (6·7) | 399 (9·6) |
| Missing, No. (%) | <10 | 305 (21·6) | 308 (7·4) |
| UT-PI at 20wk-z score (log-transformed), mean (SD) | 0·02 (1·02) | -0·06 (1) | -0·004 (1·01) |
| Frequency of high UT-PI at 20 wk, No. (%) | 287 (10·4) | 107 (7·6) | 398 (9·6) |
| Missing, No. (%) | 25 (0·9) | 88 (6·2) | 113 (2·7) |
| UMB-PI at 36 wk-z score (log-transformed), mean (SD) | 0·03 (1·02) | -0·1 (0·97) | -0·01 (1·01) |
| Frequency of high UMB-PI at 36 wk, No. (%) | 291 (10·6) | 77 (5·5) | 375 (9) |
| Missing, No. (%) | <10 | 307 (21·8) | 314 (7·5) |
| Maternal PAPP-A level at 12 wk (MoM-GA adjusted), mean (SD) | 1·15 (0·72) | 1·14 (0·74) | 1·13 (0·7) |
| Frequency of low PAPPA level (<0.4 MoM) at 12 wk, No. (%) | 101 (3·7) | 44 (3·1) | 145 (3·5) |
| Missing, No. (%) | 39 (1·4) | 93 (6·6) | 132 (3·2) |
| sFlt1:PlGF ratio at 36 wk, mean (SD) | 22·17 (31·35) | 19.46 (25·04) | 21·43 (29·76) |
| Frequency of sFlt1:PlGF ratio at 36 wk >38, No. (%) | 423 (15·4) | 137 (9·7) | 568 (13·6) |
| Missing, No. (%) | 28 (1) | 363 (25·7) | 392 (9·4) |
| AFP at 20 wk (MoM-GA adjusted), mean (SD) | 1·09 (0·35) | 1.07 (0·45) | 1·06 (0·39) |
| Frequency of AFP at 20 wk >2.0 MoM, No. (%) | 14 (0·5) | 33 (2·3) | 78 (1·9) |
| Missing, No. (%) | 40 (1·5) | 128 (9·1) | 168 (4) |

Abbreviations: AC abdominal circumference; AFP, alpha-feto protein; EFW, estimated fetal weight; PAPP-A, pregnancy associated plasma protein-A; sFlt1:PlGF, soluble fms-like tyrosine kinase 1:placental growth factor ratio; UMB-PI, umbilical artery pulsatility index; UT-PI, uterine artery pulsatility index.
