# Supplementary material for: Association between antenatal diagnosis of late fetal growth restriction and educational outcomes in mid-childhood: A UK prospective cohort study with long-term data linkage study
Source: PLoS Med. 2023 Apr 24;20(4):e1004225. doi: 10.1371/journal.pmed.1004225 (PMC10166482; doi:10.1371/journal.pmed.1004225)
Supplement: S3 Table — Values are median (IQR) or N (%) as appropriate. Maternal age was defined as age at recruitment. Maternal BMI was derived from weight measured at recruitment divided by the square of height (kg/m2). All other maternal characteristics were either self-reported at the 20-week gestational age visit, from examination of the clinical record, or linkage to the hospital’s electronic databases. Deprivation was quantified using the IMD 2007 based on census data from the area of the mother’s postcode. Birth weight percentiles and z scores were calculated using UK 1990 growth reference. Abbreviations: AGA, appropriate-for-gestational age; BMI, body mass index; FGR, fetal growth restriction; GA, gestational age; IMD, index of multiple deprivation; IQR, interquartile range; SGA, small-for-gestational age. (DOCX) [file pmed.1004225.s006.docx]

**S3 Table. Baseline characteristics among exposure groups**

|  | **FGR**  **(N=250)** | **AGA with markers of placental dysfunction (N=949)** | **Healthy SGA**  **(N=126)** | **Healthy AGA/**  **Referent**  **(N=1429)** |
| --- | --- | --- | --- | --- |
| **Maternal characteristics** | | | | |
| Age, year | 29 (25-33·8) | 30 (27-34) | 29·5 (26-33) | 30 (27-33) |
| BMI, kg/m^2^ | 22·9 (20·7-25·7) | 24·48 (22-27·6) | 23 (21·1-25·4) | 24·2 (22-27·3) |
| Ethnicity (% white) | 238 (95) | 906 (96) | 118 (94) | 1364 (96) |
| Smoking history (% never smoked) | 139 (56) | 536 (57) | 71 (56·3) | 850 (60) |
| Alcohol consumption (% not drinking) | 237 (95) | 903 (95) | 121 (96) | 1356 (95) |
| Partner status (% has partner) | 247 (99) | 930 (98) | 123 (98) | 1404 (98) |
| IMD | 9·5 (6-14·4) | 8·56 (5·7-13·7) | 8·48 (5-14·3) | 8·4 (5·5- 13·9) |
| Occupation (% management level/professional) | 121 (48) | 467 (49) | 57 (45) | 708 (50) |
| **Birth characteristics** | | | | |
| GA, weeks | 40 (39-41) | 40·1 (39-41) | 40·4 (39·6-41) | 40·6 (39·7-41·3) |
| Mode of delivery (% vaginal delivery) | 199 (80) | 696 (73) | 106 (84) | 1048 (73) |
| Sex (% female) | 139 (56) | 442 (47) | 75 (60) | 740 (52) |

Values are median (IQR) or N (%) as appropriate. Maternal age was defined as age at recruitment. Maternal BMI was derived from weight measured at recruitment divided by the square of height (kg/m^2^). All other maternal characteristics were either self-reported at the 20 week gestational age-visit, from examination of the clinical record, or linkage to the hospital’s electronic databases. Deprivation was quantified using the Index of Multiple Deprivation 2007 based on census data from the area of the mother’s postcode. Birth weight percentiles and z scores were calculated using UK 1990 growth reference.

Abbreviations: AGA, appropriate-for-gestational-age; BMI, body mass index; FGR, fetal growth restriction; GA, gestational age; IMD, index of multiple deprivation; IQR, interquartile range; SGA, small-for-gestational age
