# Supplementary material for: Association between antenatal diagnosis of late fetal growth restriction and educational outcomes in mid-childhood: A UK prospective cohort study with long-term data linkage study
Source: PLoS Med. 2023 Apr 24;20(4):e1004225. doi: 10.1371/journal.pmed.1004225 (PMC10166482; doi:10.1371/journal.pmed.1004225)
Supplement: S4 Table — Table A. Rate of passing educational standard aged 5–7 years in all exposure groups. No/total (%) of participants of each group who failed corresponding educational assessment are displayed. *p < 0.05 and **p < 0.01 versus healthy AGA (referent) based on chi-squared test. Abbreviations: AGA, appropriate-for-gestational age; FGR, fetal growth restriction; GA, gestational age. Table B. Association between educational attainment aged 5–7 years and presence of any markers of placental dysfunction by fetal growth status. Outcome: Not achieving expected educational standard at each age/domain (as appropriate). Odds ratios (OR; for unadjusted models) or adjusted odds ratios (aOR; for adjusted models) with 95% confidence intervals are displayed with antenatal healthy AGA (Total N = 1,429) as the referent group. Markers of placental dysfunction are defined as one or more of the following: low AC growth between 20–36 weeks, high uterine artery pulsatility index at 20 weeks, high umbilical artery pulsatility index at 36 weeks, EFW <third centile, low PAPPA, sFlt-1:PlGF ratio, and high AFP. For adjusted models, covariates included in all models: maternal factors (age at pregnancy, BMI at recruitment, ethnicity, occupation, partner status, smoking history), infant factors (gestational age, sex, birth seasonality, childhood physical health), socioeconomic factors (IMD, school funding type, academic year). Abbreviations: AC, abdominal circumference; AFP, alpha-feto protein; AGA, appropriate-for-gestational age; aOR, adjusted odds ratio; CI, confidence interval; EFW, estimated fetal weight; FGR, fetal growth restriction; OR, odds ratio; PAPP-A, pregnancy-associated plasma protein-A; sFlt1:PlGF, soluble fms-like tyrosine kinase 1:placental growth factor ratio; SGA, small-for-gestational age; UMB-PI, umbilical artery pulsatility index; UT-PI, uterine artery pulsatility index. (DOCX) [file pmed.1004225.s007.docx]

**S4 Table. Educational attainment aged 5-7 years by fetal growth status**

**Table A. Rate of passing educational standard aged 5-7 years in all exposure groups**

|  | **FGR** | **AGA with markers of placental dysfunction** | **Healthy SGA** | **Healthy AGA (referent)** |
| --- | --- | --- | --- | --- |
| Age 5 | 63/250 (25)* | 194/942 (21) | 28/125 (22) | 274/1418 (19) |
| Age 6 | 45/246 (18)** | 100/929 (11) | 17/125 (14) | 152/1399/ (11) |
| Age 7 - Reading | 47/223 (21)* | 122/801 (15) | 21/113 (19) | 188/1214 (15) |
| Age 7 - Writing | 62/223 (28)* | 186/802 (23) | 23/113 (20) | 257/1216 (21) |
| Age 7 - Mathematics | 53/223 (24)** | 130/802 (16) | 20/113 (18) | 196/1216 (16) |
| Age 7 - Science | 23/223 (10) | 73/802 (9) | 11/113 (10) | 122/1216 (10) |

No/total (%) of participants of each group who failed corresponding educational assessment are displayed.

*p<0·05 and **p<0·01 vs. Healthy AGA (referent) based on chi-square test

Abbreviations: AGA, appropriate-for-gestational-age; FGR, fetal growth restriction; GA, gestational age

**Table B. Association between educational attainment aged 5-7 years and presence of any markers of placental dysfunction by fetal growth status**

|  | | **FGR**  **(Total N=250)** | | **AGA with markers of placental dysfunction (Total N=949)** | | **Healthy SGA**  **(Total N=126)** | |
| --- | --- | --- | --- | --- | --- | --- | --- |
|  |  | OR (95% CI) | p | OR (95% CI) | p | OR (95% CI) | p |
| Age 5 | *Unadjusted* | 1·41 (1·02-1·92) | 0·03 | 1·08 (0·88-1·33) | 0·45 | 1·21 (0·76-1·85) | 0·41 |
|  | *Adjusted* | 1·33 (0·93-1·89) | 0·11 | 0·96 (0·77-1·21) | 0·74 | 1·41 (0·86-2·25) | 0·16 |
| Age 6 | *Unadjusted* | 1·84 (1·26-2·63) | 0·001 | 0·99 (0·76-1·29) | 0·94 | 1·29 (0·73-2·16) | 0·35 |
|  | *Adjusted* | 1·68 (1·12-2·48) | 0·01 | 0·88 (0·66-1·17) | 0·38 | 1·4 (0·77-2·42) | 0·24 |
| Age 7 - Reading | *Unadjusted* | 1·46 (1·01-2·07) | 0·04 | 0·98 (0·76-1·25) | 0·88 | 1·25 (0·74-2·01) | 0·39 |
|  | *Adjusted* | 1·46 (0·99-2·13) | 0·05 | 0·93 (0·71-1·2) | 0·57 | 1·41 (0·82-2·35) | 0·2 |
| Age 7 - Writing | *Unadjusted* | 1·44 (1·03-1·98) | 0·03 | 1·13 (0·91-1·39) | 0·27 | 0·95 (0·58-1·51) | 0·85 |
|  | *Adjusted* | 1·46 (1·01-2·07) | 0·04 | 0·98 (0·76-1·25) | 0·88 | 1·25 (0·74-2·01) | 0·39 |
| Age 7 -Mathematics | *Unadjusted* | 1·62 (1·14-2·28) | 0·006 | 1·01 (0·79-1·28) | 0·96 | 1·12 (0·66-1·82) | 0·66 |
|  | *Adjusted* | 1·49 (1·02-2·15) | 0·03 | 0·95 (0·73-1·23) | 0·69 | 1·15 (0·66-1·93) | 0·61 |
| Age 7 - Science | *Unadjusted* | 1·03 (0·63-1·62) | 0·9 | 0·9 (0·66-1·21) | 0·49 | 0·97 (0·48-1·78) | 0·92 |
|  | *Adjusted* | 0·98 (0·58-1·58) | 0·92 | 0·86 (0·63-1·19) | 0·37 | 1·07 (0·52-2·02) | 0·84 |

Outcome: Not achieving expected educational standard at each age/domain (as appropriate)

Odds ratios (OR; for unadjusted models) or adjusted odds ratios (aOR; for adjusted models) with 95% confidence intervals are displayed with antenatal healthy AGA (Total N=1429) as the referent group.

Markers of placental dysfunction are defined as one or more of the following: low AC growth between 20-36 weeks, high uterine artery pulsatility index at 20 weeks, high umbilical artery pulsatility index at 36 weeks, EFW <3rd centile, low PAPPA, sFlt-1:PlGF ratio, high AFP.

For adjusted models, covariates included in all models: maternal factors (age at pregnancy, BMI at recruitment, ethnicity, occupation, partner status, smoking history), infant factors (gestational age, sex, birth seasonality, childhood physical health), socio-economic factors (IMD, school funding type, academic year).

Abbreviations: AC, abdominal circumference; AFP, alpha-feto protein; AGA, appropriate-for-gestational-age; aOR, adjusted odds ratio; CI, confidence interval; EFW, estimated fetal weight; FGR, fetal growth restriction; OR, odds ratio; PAPP-A, pregnancy-associated plasma protein-A; sFlt1:PlGF, soluble fms-like tyrosine kinase 1:placental growth factor ratio; SGA, small-for-gestational-age ; UMB-PI, umbilical artery pulsatility index; UT-PI, uterine artery pulsatility index
