# Supplementary material for: Association between antenatal diagnosis of late fetal growth restriction and educational outcomes in mid-childhood: A UK prospective cohort study with long-term data linkage study
Source: PLoS Med. 2023 Apr 24;20(4):e1004225. doi: 10.1371/journal.pmed.1004225 (PMC10166482; doi:10.1371/journal.pmed.1004225)
Supplement: S5 Table — Table A. Association between educational attainment aged 5 and markers of placental dysfunction by fetal growth status. Outcome: Not achieving expected educational standard aged 5. Odds ratios (OR) with 95% confidence intervals are displayed with antenatal healthy AGA (N = 1,418) as the referent group. aLow AC velocity, High UT-PI, High UMB-PI, EFW 38, High AFP; bLow AC velocity, High UT-PI, High UMB-PI, EFW 38, High AFP. Covariates included in fully adjusted models: maternal factors (age at pregnancy, BMI at recruitment, ethnicity, occupation, partner status, smoking history), infant factors (gestational age, sex, birth seasonality, childhood physical health), socioeconomic factors (IMD, school funding type, academic year). Abbreviations: AC, abdominal circumference; AFP, alpha-feto protein; AGA, appropriate-for-gestational-age; aOR, adjusted odds ratio; CI, confidence interval; EFW, estimated fetal weight; FGR, fetal growth restriction; NA, not applicable; PAPP-A, pregnancy-associated plasma protein-A; sFlt1:PlGF, soluble fms-like tyrosine kinase 1:placental growth factor ratio; SGA, small-for-gestational-age; UMB-PI, umbilical artery pulsatility index; UT-PI, uterine artery pulsatility index. Table B. Association between educational attainment aged 6 and markers of placental dysfunction by fetal growth status. Outcome: Not achieving expected educational standard aged 6. Odds ratios (OR) with 95% confidence intervals are displayed with antenatal healthy AGA (N = 1,399) as the referent group. aLow AC velocity, High UT-PI, High UMB-PI, EFW 38, High AFP; bLow AC velocity, High UT-PI, High UMB-PI, EFW 38, High AFP. Covariates included in fully adjusted models: maternal factors (age at pregnancy, BMI at recruitment, ethnicity, occupation, partner status, smoking history), infant factors (gestational age, s [file pmed.1004225.s008.docx]

**S5 Table. Associations between educational attainment aged 5-7 and markers of placental dysfunction by fetal growth status**

**Table A. Association between educational attainment aged 5 and markers of placental dysfunction by fetal growth status**

|  | **FGR**  **(N=250)** | | **AGA with markers of**  **placental dysfunction**  **(N=942)** | | **Healthy SGA**  **(N=125)** | |
| --- | --- | --- | --- | --- | --- | --- |
|  | OR (95% CI) | p | OR (95% CI) | p | OR (95% CI) | p |
| Unadjusted models | | | | | | |
| All markers^a^ | 1·41 (1·02-1·92) | 0·03 | 1·08 (0·88-1·33) | 0·45 | 1·21 (0·76-1·85) | 0·41 |
| Scan markers only^b^ | 1·34 (0·94-1·87) | 0·09 | 1·04 (0·81-1·32) | 0·76 | 1·21 (0·76-1·85) | 0·41 |
| Biomarkers only^c^ | 1·66 (1·04-2·57) | 0·03 | 1·12 (0·87-1·44) | 0·38 | 1·21 (0·76-1·85) | 0·41 |
| Fully adjusted models | | | | | | |
| All markers^a^ | 1·33 (0·93-1·89) | 0·11 | 0·96 (0·77-1·21) | 0·74 | 1·41 (0·86-2·25) | 0·16 |
| Scan markers only^b^ | 1·27 (0·85-1·85) | 0·23 | 0·97 (0·74-1·27) | 0·85 | 1·43 (0·87-2·29) | 0·15 |
| Biomarkers only^c^ | 1·47 (0·87-2·42) | 0·14 | 0·99 (0·74-1·31) | 0·94 | 1·4 (0·85-2·24) | 0·17 |

Outcome: Not achieving expected educational standard aged 5

Odds ratios (OR) with 95% confidence intervals are displayed with antenatal healthy AGA (N=1418) as the referent group.

^a^Low AC velocity, High UT-PI , High UMB-PI, EFW <3^rd^ centile, Low PAPP-A, sFlt-1:PlGF >38, High AFP

^b^Low AC velocity, High UT-PI , High UMB-PI, EFW <3^rd^ centile

^c^Low PAPP-A, sFlt-1:PlGF >38, High AFP

Covariates included in fully adjusted models: maternal factors (age at pregnancy, BMI at recruitment, ethnicity, occupation, partner status, smoking history), infant factors (gestational age, sex, birth seasonality, childhood physical health), socio-economic factors (IMD, school funding type, academic year)

Abbreviations: AC, abdominal circumference; AFP, alpha-feto protein; AGA, appropriate-for-gestational-age; aOR, adjusted odds ratio; CI, confidence interval; EFW, estimated fetal weight; FGR, fetal growth restriction; NA, not applicable; PAPP-A, pregnancy-associated plasma protein-A; sFlt1:PlGF, soluble fms-like tyrosine kinase 1:placental growth factor ratio; SGA, small-for-gestational-age ; UMB-PI, umbilical artery pulsatility index; UT-PI, uterine artery pulsatility index

**Table B. Association between educational attainment aged 6 and markers of placental dysfunction by fetal growth status**

|  | **FGR**  **(N=246)** | | **AGA with markers of**  **placental dysfunction**  **(N=929)** | | **Healthy SGA**  **(N=125)** | |
| --- | --- | --- | --- | --- | --- | --- |
|  | OR (95% CI) | p | OR (95% CI) | p | OR (95% CI) | p |
| Unadjusted models | | | | | | |
| All markers^a^ | 1·84 (1·26-2·63) | 0·001 | 0·99 (0·76-1·29) | 0·94 | 1·29 (0·73-2·16) | 0·35 |
| Scan markers only^b^ | 1·73 (1·15-2·54) | 0·007 | 0·97 (0·71-1·32) | 0·86 | 1·29 (0·73-2·16) | 0·35 |
| Biomarkers only^c^ | 2·03 (1·18-3·34) | 0·008 | 1·08 (0·77-1·48) | 0·66 | 1·29 (0·73-2·16) | 0·35 |
| Fully adjusted models | | | | | | |
| All markers^a^ | 1·68 (1·12-2·48) | 0·01 | 0·88 (0·66-1·17) | 0·38 | 1·4 (0·77-2·42) | 0·24 |
| Scan markers only^b^ | 1·61 (1·03-2·48) | 0·03 | 0·87 (0·62-1·21) | 0·42 | 1·43 (0·78-2·48) | 0·23 |
| Biomarkers only^c^ | 1·63 (0·91-2·83) | 0·09 | 0·95 (0·67-1·34) | 0·77 | 1·35 (0·74-2·34) | 0·31 |

Outcome: Not achieving expected educational standard aged 6

Odds ratios (OR) with 95% confidence intervals are displayed with antenatal healthy AGA (N=1399) as the referent group.

^a^Low AC velocity, High UT-PI , High UMB-PI, EFW <3^rd^ centile, Low PAPP-A, sFlt-1:PlGF >38, High AFP

^b^Low AC velocity, High UT-PI , High UMB-PI, EFW <3^rd^ centile

^c^Low PAPP-A, sFlt-1:PlGF >38, High AFP

Covariates included in fully adjusted models: maternal factors (age at pregnancy, BMI at recruitment, ethnicity, occupation, partner status, smoking history), infant factors (gestational age, sex, birth seasonality, childhood physical health), socio-economic factors (IMD, school funding type, academic year)

Abbreviations: AC, abdominal circumference; AFP, alpha-feto protein; AGA, appropriate-for-gestational-age; aOR, adjusted odds ratio; CI, confidence interval; EFW, estimated fetal weight; FGR, fetal growth restriction; NA, not applicable; PAPP-A, pregnancy-associated plasma protein-A; sFlt1:PlGF, soluble fms-like tyrosine kinase 1:placental growth factor ratio; SGA, small-for-gestational-age ; UMB-PI, umbilical artery pulsatility index; UT-PI, uterine artery pulsatility index

**Table C. Association between educational attainment aged 7 (Reading domain) and markers of placental dysfunction by fetal growth status**

|  | **FGR**  **(N=223)** | | **AGA with markers of**  **placental dysfunction**  **(N=801)** | | **Healthy SGA**  **(N=113)** | |
| --- | --- | --- | --- | --- | --- | --- |
|  | OR (95% CI) | p | OR (95% CI) | p | OR (95% CI) | p |
| Unadjusted models | | | | | | |
| All markers^a^ | 1·46 (1·01-2·07) | 0·04 | 0·98 (0·76-1·25) | 0·88 | 1·25 (0·74-2·01) | 0·39 |
| Scan markers only^b^ | 1·3 (0·87-1·91) | 0·19 | 1·03 (0·77-1·37) | 0·83 | 1·25 (0·74-2·01) | 0·39 |
| Biomarkers only^c^ | 1·87 (1·13-3·01) | 0·01 | 0·95 (0·69-1·28) | 0·73 | 1·25 (0·74-2·01) | 0·39 |
| Fully adjusted models | | | | | | |
| All markers^a^ | 1·46 (0·99-2·13) | 0·05 | 0·93 (0·71-1·2) | 0·57 | 1·41 (0·82-2·35) | 0·2 |
| Scan markers only^b^ | 1·33 (0·86-2·01) | 0·2 | 1·0 (0·73-1·35) | 0·99 | 1·42 (0·82-2·37) | 0·19 |
| Biomarkers only^c^ | 1·82 (1·05-3·07) | 0·03 | 0·89 (0·63-1·23) | 0·47 | 1·4 (0·81-2·32) | 0·21 |

Outcome: Not achieving expected educational standard aged 7 in Reading domain

Odds ratios (OR) with 95% confidence intervals are displayed with antenatal healthy AGA (N=1214) as the referent group.

^a^Low AC velocity, High UT-PI , High UMB-PI, EFW <3^rd^ centile, Low PAPP-A, sFlt-1:PlGF >38, High AFP

^b^Low AC velocity, High UT-PI , High UMB-PI, EFW <3^rd^ centile

^c^Low PAPP-A, sFlt-1:PlGF >38, High AFP

Covariates included in all models: maternal factors (age at pregnancy, BMI at recruitment, ethnicity, occupation, partner status, smoking history), infant factors (GA, sex, birth seasonality, childhood physical health), socio-economic factors (IMD, school funding type, academic year)

Abbreviations: AC, abdominal circumference; AFP, alpha-feto protein; AGA, appropriate-for-gestational-age; aOR, adjusted odds ratio; CI, confidence interval; EFW, estimated fetal weight; FGR, fetal growth restriction; NA, not applicable; PAPP-A, pregnancy-associated plasma protein-A; sFlt1:PlGF, soluble fms-like tyrosine kinase 1:placental growth factor ratio; SGA, small-for-gestational-age; UMB-PI, umbilical artery pulsatility index; UT-PI, uterine artery pulsatility index

**Table D. Association between educational attainment aged 7 (Writing domain) and markers of placental dysfunction by fetal growth status**

|  | **FGR**  **(N=223)** | | **AGA with markers of**  **placental dysfunction**  **(N=802)** | | **Healthy SGA**  **(N=113)** | |
| --- | --- | --- | --- | --- | --- | --- |
|  | OR (95% CI) | p | OR (95% CI) | p | OR (95% CI) | p |
| Unadjusted models | | | | | | |
| All markers^a^ | 1·44 (1·03-1·98) | 0·03 | 1·13 (0·91-1·39) | 0·27 | 0·95 (0·58-1·51) | 0·85 |
| Scan markers only^b^ | 1·4 (0·98-1·97) | 0·06 | 1·15 (0·89-1·47) | 0·28 | 0·95 (0·58-1·51) | 0·85 |
| Biomarkers only^c^ | 1·66 (1·04-2·61) | 0·03 | 1·11 (0·85-1·45) | 0·42 | 0·95 (0·58-1·51) | 0·85 |
| Fully adjusted models | | | | | | |
| All markers^a^ | 1·46 (1·02-2·07) | 0·04 | 1·06 (0·84-1·33) | 0·62 | 1·09 (0·64-1·78) | 0·74 |
| Scan markers only^b^ | 1·46 (0·99-2·14) | 0·05 | 1·12 (0·85-1·46) | 0·42 | 1·09 (0·64-1·78) | 0·73 |
| Biomarkers only^c^ | 1·55 (0·93-2·55) | 0·09 | 1·02 (0·76-1·35) | 0·9 | 1·09 (0·64-1·78) | 0·74 |

Outcome: Not achieving expected educational standard aged 7 in Writing domain

Odds ratios (OR) with 95% confidence intervals are displayed with antenatal healthy AGA (N=1216) as the referent group.

^a^Low AC velocity, High UT-PI , High UMB-PI, EFW <3^rd^ centile, Low PAPP-A, sFlt-1:PlGF >38, High AFP

^b^Low AC velocity, High UT-PI , High UMB-PI, EFW <3^rd^ centile

^c^Low PAPP-A, sFlt-1:PlGF >38, High AFP

Covariates included in all models: maternal factors (age at pregnancy, BMI at recruitment, ethnicity, occupation, partner status, smoking history), infant factors (GA, sex, birth seasonality, childhood physical health), socio-economic factors (IMD, school funding type, academic year)

Abbreviations: AC, abdominal circumference; AFP, alpha-feto protein; AGA, appropriate-for-gestational-age; aOR, adjusted odds ratio; CI, confidence interval; EFW, estimated fetal weight; FGR, fetal growth restriction; NA, not applicable; PAPP-A, pregnancy-associated plasma protein-A; sFlt1:PlGF, soluble fms-like tyrosine kinase 1:placental growth factor ratio; SGA, small-for-gestational-age; UMB-PI, umbilical artery pulsatility index; UT-PI, uterine artery pulsatility index

**Table E. Association between educational attainment aged 7 (Mathematics domain) and markers of placental dysfunction by fetal growth status**

|  | **FGR**  **(N=223)** | | **AGA with markers of**  **placental dysfunction**  **(N=802)** | | **Healthy SGA**  **(N=113)** | |
| --- | --- | --- | --- | --- | --- | --- |
|  | OR (95% CI) | p | OR (95% CI) | p | OR (95% CI) | p |
| Unadjusted models | | | | | | |
| All markers^a^ | 1·62 (1·14-2·28) | 0·006 | 1·01 (0·79-1·28) | 0·96 | 1·12 (0·66-1·82) | 0·66 |
| Scan markers only^b^ | 1·6 (1·1-2·3) | 0·01 | 1·03 (0·77-1·36) | 0·86 | 1·12 (0·66-1·82) | 0·66 |
| Biomarkers only^c^ | 2·1 (1·29-3·33) | 0·002 | 1·04 (0·77-1·4) | 0·79 | 1·12 (0·66-1·82) | 0·66 |
| Fully adjusted models | | | | | | |
| All markers^a^ | 1·49 (1·02-2·15) | 0·03 | 0·95 (0·73-1·23) | 0·69 | 1·15 (0·66-1·93) | 0·61 |
| Scan markers only^b^ | 1·5 (0·99-2·22) | 0·05 | 0·99 (0·73-1·33) | 0·97 | 1·13 (0·65-1·9) | 0·65 |
| Biomarkers only^c^ | 1·78 (1·05-2·96) | 0·03 | 1·0 (0·72-1·37) | >0·99 | 1·15 (0·66-1·94) | 0·61 |

Outcome: Not achieving expected educational standard aged 7 in Mathematics domain

Odds ratios (OR) with 95% confidence intervals are displayed with antenatal healthy AGA (N=1216) as the referent group.

^a^Low AC velocity, High UT-PI , High UMB-PI, EFW <3^rd^ centile, Low PAPP-A, sFlt-1:PlGF >38, High AFP

^b^Low AC velocity, High UT-PI , High UMB-PI, EFW <3^rd^ centile

^c^Low PAPP-A, sFlt-1:PlGF >38, High AFP

Covariates included in all models: maternal factors (age at pregnancy, BMI at recruitment, ethnicity, occupation, partner status, smoking history), infant factors (GA, sex, birth seasonality, childhood physical health), socio-economic factors (IMD, school funding type, academic year)

Abbreviations: AC, abdominal circumference; AFP, alpha-feto protein; AGA, appropriate-for-gestational-age; aOR, adjusted odds ratio; CI, confidence interval; EFW, estimated fetal weight; FGR, fetal growth restriction; NA, not applicable; PAPP-A, pregnancy-associated plasma protein-A; sFlt1:PlGF, soluble fms-like tyrosine kinase 1:placental growth factor ratio; SGA, small-for-gestational-age; UMB-PI, umbilical artery pulsatility index; UT-PI, uterine artery pulsatility index

**Table F. Association between educational attainment aged 7 (Science domain) and markers of placental dysfunction by fetal growth status**

|  | **FGR**  **(N=223)** | | **AGA with markers of**  **placental dysfunction**  **(N=802)** | | **Healthy SGA**  **(N=113)** | |
| --- | --- | --- | --- | --- | --- | --- |
|  | OR (95% CI) | p | OR (95% CI) | p | OR (95% CI) | p |
| Unadjusted models | | | | | | |
| All markers^a^ | 1·03 (0·63-1·62) | 0·9 | 0·9 (0·66-1·21) | 0·49 | 0·97 (0·48-1·78) | 0·92 |
| Scan markers only^b^ | 0·93 (0·51-1·49) | 0·69 | 0·93 (0·65-1·32) | 0·71 | 0·97 (0·48-1·78) | 0·92 |
| Biomarkers only^c^ | 1·19 (0·58-2·2) | 0·61 | 0·9 (0·61-1·31) | 0·6 | 0·97 (0·48-1·78) | 0·92 |
| Fully adjusted models | | | | | | |
| All markers^a^ | 0·98 (0·58-1·58) | 0·92 | 0·86 (0·63-1·19) | 0·37 | 1·07 (0·52-2·02) | 0·84 |
| Scan markers only^b^ | 0·85 (0·47-1·45) | 0·56 | 0·89 (0·61-1·29) | 0·55 | 1·07 (0·52-2·02) | 0·85 |
| Biomarkers only^c^ | 1·09 (0·52-2·12) | 0·81 | 0·9 (0·6-1·35) | 0·63 | 1·07 (0·52-2·02) | 0·85 |

Outcome: Not achieving expected educational standard aged 7 in Science domain

Odds ratios (OR) with 95% confidence intervals are displayed with antenatal healthy AGA (N=1216) as the referent group.

^a^Low AC velocity, High UT-PI , High UMB-PI, EFW <3^rd^ centile, Low PAPP-A, sFlt-1:PlGF >38, High AFP

^b^Low AC velocity, High UT-PI , High UMB-PI, EFW <3^rd^ centile

^c^Low PAPP-A, sFlt-1:PlGF >38, High AFP

Covariates included in all models: maternal factors (age at pregnancy, BMI at recruitment, ethnicity, occupation, partner status, smoking history), infant factors (GA, sex, birth seasonality, childhood physical health), socio-economic factors (IMD, school funding type, academic year)

Abbreviations: AC, abdominal circumference; AFP, alpha-feto protein; AGA, appropriate-for-gestational-age; aOR, adjusted odds ratio; CI, confidence interval; EFW, estimated fetal weight; FGR, fetal growth restriction; NA, not applicable; PAPP-A, pregnancy-associated plasma protein-A; sFlt1:PlGF, soluble fms-like tyrosine kinase 1:placental growth factor ratio; SGA, small-for-gestational-age; UMB-PI, umbilical artery pulsatility index; UT-PI, uterine artery pulsatility index
