# Supplementary material for: Association between antenatal diagnosis of late fetal growth restriction and educational outcomes in mid-childhood: A UK prospective cohort study with long-term data linkage study
Source: PLoS Med. 2023 Apr 24;20(4):e1004225. doi: 10.1371/journal.pmed.1004225 (PMC10166482; doi:10.1371/journal.pmed.1004225)
Supplement: S6 Table — Outcome: Not achieving expected educational standard at each corresponding age. Adjusted odds ratios (OR) with 95% confidence intervals of FGR are displayed with healthy AGA as the referent group. P values are based on on logistic regression models of educational performance between 4 antenatal exposure groups: (1) Antenatal FGR; (2) Antenatal healthy SGA; (3) Antenatal AGA with markers of placental dysfunction; and (4) Antenatal healthy AGA. Models are stratified into infants who were SGA vs. AGA at birth. All models are adjusted for the following: maternal factors (age at pregnancy, BMI at recruitment, ethnicity, occupation, partner status, smoking history), infant factors (gestational age, sex, birth seasonality, childhood physical health), socioeconomic factors (IMD, school funding, academic year). Markers of placental dysfunction are defined as one or more of the following: low AC growth between 20–36 weeks, high UT-PI at 20 weeks, high UMB-PI at 36 weeks, EFW <third centile, low PAPPA, sflt1:PlGF ratio, and high AFP. Abbreviations: AC, abdominal circumference; AFP, alpha-feto protein; AGA, appropriate-for-gestational age; aOR, adjusted odds ratio; CI, confidence interval; EFW, estimated fetal weight; FGR, fetal growth restriction; PAPP-A, pregnancy-associated plasma protein-A; sFlt1:PlGF, soluble fms-like tyrosine kinase 1:placental growth factor ratio; SGA, small-for-gestational age; UMB-PI, umbilical artery pulsatility index; UT-PI, uterine artery pulsatility index. (DOCX) [file pmed.1004225.s009.docx]

**S6 Table. Association between FGR and educational attainment aged 5-7, stratified based on actual birth weight**

|  | **≥10^th^ centile at birth**  **(Antenatal FGR)** | | **<10^th^ centile at birth**  **(Antenatal FGR)** | |
| --- | --- | --- | --- | --- |
|  | **aOR (95% CI)** | ***p*** | **aOR (95% CI)** | ***p*** |
| **Age 5** | 0·99 (0·61-1·55) | 0·95 | 2·4 (0·79-8·04) | 0·14 |
| **Age 6** | 1·34 (0·79-2·21) | 0·26 | 6·62 (1·61-37·02) | 0·02 |
| **Reading Age 7** | 1·2 (0·72-1·95) | 0·46 | 2·56 (0·75-10·1) | 0·15 |
| **Writing Age 7** | 1·3 (0·82-2·01) | 0·25 | 3·27 (1·0-12·08) | 0·06 |
| **Mathematics Age 7** | 1·16 (0·7-1·86) | 0·56 | 1·71 (0·57-5·53) | 0·35 |
| **Science Age 7** | 0·82 (0·41-1·51) | 0·54 | 1·1 (0·21-6·78) | 0·91 |

Outcome: Not achieving expected educational standard at each corresponding age.

Adjusted odds ratios (OR) with 95% confidence intervals of FGR are displayed with healthy AGA as the referent group.

P values are based on on logistic regression models of educational performance between 4 antenatal exposure groups: (1) Antenatal FGR, (2) Antenatal healthy SGA, (3) Antenatal AGA with markers of placental dysfunction, and (4) Antenatal healthy AGA. Models are stratified into infants who were SGA vs AGA at birth.

All models are adjusted for: maternal factors (age at pregnancy, BMI at recruitment, ethnicity, occupation, partner status, smoking history), infant factors (gestational age, sex, birth seasonality, childhood physical health), socio-economic factors (IMD, school funding, academic year)

Markers of placental dysfunction are defined as one or more of the following: low AC growth between 20-36 weeks, high uterine artery pulsatility index at 20 weeks, high umbilical artery pulsatility index at 36 weeks, EFW <3rd centile, low PAPPA, sflt1:PlGF ratio, and high AFP
