# Supplementary material for: Association between antenatal diagnosis of late fetal growth restriction and educational outcomes in mid-childhood: A UK prospective cohort study with long-term data linkage study
Source: PLoS Med. 2023 Apr 24;20(4):e1004225. doi: 10.1371/journal.pmed.1004225 (PMC10166482; doi:10.1371/journal.pmed.1004225)
Supplement: S8 Table — Outcome: Not achieving expected educational standard at each corresponding age. Adjusted odds ratios (OR) with 95% confidence intervals of FGR are displayed with healthy AGA as the referent group. *P values < 0.05, based on logistic regression models of educational performance between 4 antenatal exposure groups: (1) Antenatal FGR; (2) Antenatal healthy SGA; (3) Antenatal AGA with markers of placental dysfunction; and (4) Antenatal healthy AGA. All models are adjusted for the following: maternal factors (age at pregnancy, BMI at recruitment, ethnicity, occupation, partner status, smoking history), infant factors (gestational age, sex, birth seasonality, childhood physical health), socioeconomic factors (IMD, school funding, academic year). Markers of placental dysfunction are defined as one or more of the following: low AC growth between 20–36 weeks, high uterine artery pulsatility index at 20 weeks, high umbilical artery pulsatility index at 36 weeks, EFW <third centile, low PAPPA, sflt1:PlGF ratio, and high AFP. (DOCX) [file pmed.1004225.s011.docx]

**S8 Table. Sensitivity analysis comparing full cohort vs cohort without subjects with clinically indicated scans at >35 weeks of gestation when associating antenatal late FGR and educational attainment aged 5-7**

| **Assessment** | **Full cohort** | **Excluding clinically indicated scans at**  **>35 weeks of gestation** |
| --- | --- | --- |
|  | **(N=250)** | **(N=152)** |
| **Age 5** | 1.33 (0.93-1.89) | 1.27 (0.81-1.96) |
| **Age 6** | 1.68 (1.12-2.48)* | 1.38 (0.81-2.28) |
| **Age 7** |  |  |
| **Reading** | 1.46 (0.99-2.13)* | 1.33 (0.80-2.14) |
| **Writing** | 1.46 (1.02-2.07)* | 1.52 (0.97-2.34) |
| **Mathematics** | 1.49 (1.02-2.15)* | 1.51 (0.93-2.38) |
| **Science** | 0.98 (0.58-1.58) | 1.06 (0.56-1.91) |

Outcome: Not achieving expected educational standard at each corresponding age.

Adjusted odds ratios (OR) with 95% confidence intervals of FGR are displayed with healthy AGA as the referent group.

*P values<0.05, based on on logistic regression models of educational performance between 4 antenatal exposure groups: (1) Antenatal FGR, (2) Antenatal healthy SGA, (3) Antenatal AGA with markers of placental dysfunction, and (4) Antenatal healthy AGA.

All models are adjusted for: maternal factors (age at pregnancy, BMI at recruitment, ethnicity, occupation, partner status, smoking history), infant factors (gestational age, sex, birth seasonality, childhood physical health), socio-economic factors (IMD, school funding, academic year)

Markers of placental dysfunction are defined as one or more of the following: low AC growth between 20-36 weeks, high uterine artery pulsatility index at 20 weeks, high umbilical artery pulsatility index at 36 weeks, EFW <3rd centile, low PAPPA, sflt1:PlGF ratio, and high AFP
