# Supplementary material for: Association between antenatal diagnosis of late fetal growth restriction and educational outcomes in mid-childhood: A UK prospective cohort study with long-term data linkage study
Source: PLoS Med. 2023 Apr 24;20(4):e1004225. doi: 10.1371/journal.pmed.1004225 (PMC10166482; doi:10.1371/journal.pmed.1004225)
Supplement: Attachment — Submitted filename: Laurentya_Response to reviewers.docx [file pmed.1004225.s012.docx]

20/02/2023

Dear Dr Davidson,

Thank you for the opportunity to revise our paper for further consideration.

We are grateful to the reviewers and the Associate Editor for their thoughtful reviews and comments, which have improved the clarity of our manuscript. We have given careful consideration to all the points raised and addressed these in our revision.

Best wishes,

Prof Gordon C S Smith
Head of Department of Obstetrics & Gynaecology, University of Cambridge

Dr Catherine Aiken

Associate Professor and Honorary Consultant in Maternal and Fetal Medicine

University of Cambridge, UK

Please revise your title according to PLOS Medicine's style. Please place the study design ("A cohort study”) in the subtitle (ie, after a colon)

**We have now added the study design in the title (“A prospective cohort study with long-term data-linkage study”).**

In the last sentence of the Abstract Methods and Findings section, please describe the main limitation(s) of the study's methodology.

**We have now added the main limitation of this study in the Abstract (Lines 45-50).**

The Data Availability Statement (DAS) requires revision. For each data source used in your study:
a) If the data are owned by a third party but freely available upon request, please note this and state the owner of the data set and contact information for data requests (web or email address). Note that a study author cannot be the contact person for the data.
b) If the data are not freely available, please describe briefly the ethical, legal, or contractual restriction that prevents you from sharing it. Please also include an appropriate contact (web or email address) for inquiries (again, this cannot be a study author).

**We have added a revised DAS statement to the manuscript (Lines 399-409).**

Thank you for including an Author Summary. The third bullet point under ‘Why was this study done’ appears better suited to ‘What did the researchers do and find?’.

**We have edited this accordingly (Author Summary – page 4).**

Please describe and cite your Supporting Information items as outlined here: <https://eur03.safelinks.protection.outlook.com/?url=https%3A%2F%2Fjournals.plos.org%2Fplosmedicine%2Fs%2Fsupporting-information%23loc-item-description&data=05%7C01%7Ccema2%40universityofcambridgecloud.onmicrosoft.com%7C8eb34a0b02844208a6f808db05301ea3%7C49a50445bdfa4b79ade3547b4f3986e9%7C1%7C0%7C638109476452112416%7CUnknown%7CTWFpbGZsb3d8eyJWIjoiMC4wLjAwMDAiLCJQIjoiV2luMzIiLCJBTiI6Ik1haWwiLCJXVCI6Mn0%3D%7C3000%7C%7C%7C&sdata=H3TAcxIS8329XA%2BImP7lFdS%2B1OKC793JsdpplUrN7sI%3D&reserved=0>

**We have edited this throughout the manuscript and supplementary materials.**

Line 209: For consistency, please place numerators/denominators within and percentages outside of parentheses.

**We have edited this accordingly.**

Please remove the Competing interests, Funding, and Author contributions from the main text – all are captured as metadata via the submission form questionnaire. The acknowledgements can be kept in the main text.

**These have been removed as advised.**

Please add to the competing interests (submission form): GS serves on PLOS Medicine’s editorial board.

**We will add this to the competing interests form during submission.**

For [Internet] sources in the References, please include the date accessed.

**We have now added dates of access for internet sources in the references.**

Comments from the reviewers:

Reviewer #1: Alex McConnachie, Statistical Review

This review considers the statistical aspects of the paper by Olga et al, looking at fetal growth restriction and measures of educational attainment at age 5-7.

The basic approach of using logistic regression, with adjustment for potential confounders, to estimate associations between the exposures of interest and failure to achieve the expected educational milestones is reasonable, though by dichotomized the outcomes, there is a risk that the study is losing power. It could have been more efficient to look at the outcomes as continuous measures, though this could be difficult, depending on the distribution of the outcomes.

**Thank you; we understand the rationale for this comment. However the scoring of the national educational assessments (described in detail in S2 Appendix) does not correspond to true continuous outcomes. The actual scores can take a limited range of values and have significantly skewed distributions.**

**Moreover the raw individual pupil scores (which are generated at school level and reported centrally to the Department for Education) are processed via a national bench-marking system to obtain the binary variables indicating whether the child has attained the expected level or not (details of this process are laid out in S2 Appendix). Our pre-defined analysis plan specified these binary outcomes at each age as the primary study outcome to ensure that a robust and validated national metric that is free of bias was used, rather than attempting to derive and interpret any study-specific metrics from the raw scores.**

**However, we do have access to the raw score data on test results aged 5 and 6 (these are not reported centrally at age 7), which we have included here (Table 1 below) for interest. These give essentially the same results as the binary outcome measures, which is reassuring with regard to the robustness of the analysis. However, as they do not further advance understanding and could introduce confusion in interpretation, we have not currently added them to the manuscript, preferring instead the pre-defined standardised binary outcomes.**

**Table 1 Comparison of numerical educational assessment scores aged 5-6 years across groups**

|  |  | **FGR**  **(Total N=250)** | | **AGA with markers of placental dysfunction (Total N=949)** | | **Healthy SGA**  **(Total N=126)** | | **Healthy AGA (referent;**  **Total N=1429)** |
| --- | --- | --- | --- | --- | --- | --- | --- | --- |
|  |  | **Median(IQR)** | **p** | **Median(IQR)** | **p** | **Median(IQR)** | **p** | **Median(IQR)** |
| **Age 5** | **Raw scores** | **34 (6)** | **.002** | **36 (7)** | **.16** | **36 (6)** | **.61** | **36 (7)** |
|  | **Z-scores** | **-0.24 (1.11)** | **.01** | **-0.09 (1.06)** | **.34** | **-0.07 (1.06)** | **.7** | **-0.09 (1.11)** |
|  | **MoM** | **0.97 (0.21)** | **.005** | **1 (0.19)** | **.73** | **1 (0.19)** | **.26** | **1 (0.19)** |
|  | **Mean of MoM*** | **1.0 (0.19)** |  | **1.02 (0.18)** |  | **1.01 (0.19)** |  | **1.03 (0.18)** |
| **Age 6** | **Raw scores** | **36 (6)** | **<.001** | **37 (4)** | **.69** | **37 (5)** | **.36** | **37 (4)** |
|  | **Z-scores** | **0.1 (0.9)** | **.003** | **0.37 (0.63)** | **.6** | **0.24 (0.79)** | **.42** | **0.26 (0.68)** |
|  | **MoM** | **0.97 (0.16)** | **.002** | **1 (0.11)** | **.53** | **1 (0.14)** | **.47** | **1 (0.11)** |
|  | **Mean of MoM*** | **0.93 (0.18)** |  | **0.96 (0.17)** |  | **0.95 (0.17)** |  | **0.96 (0.17)** |

**Both EY/phonics z-scores and MoM are adjusted for academic years. Phonics results were based on recorded outcomes on the first attempts.**

***Values are mean(SD)**

**Significant p values are typed in bold. Significance is assessed between each exposure group vs. healthy AGA as the referent separately using non-parametric Mann-Whitney U test.**

**Abbreviations: AGA, appropriate-for-gestational-age; EY, early years; FGR, fetal growth restriction; MoM, multiple of the median; SGA, small-for-gestational-age**

The main issue with the analysis is that the authors have taken two separate exposures (small for gestational age, and placental dysfunction) and combined these to produce a single variable with 4 levels. They then look at each of the three subgroups with one or both exposures, and compare them to the subgroup with neither exposure. They interpret a "significant" p-value as giving evidence of an association, and a "non-significant" p-value as meaning that the outcomes were comparable. This is a false argument.

For example, looking at supplementary table 4B, at age 7, the adjusted OR for reading for FGR vs Healthy AGA is 1.46, p=0.05, whereas for Health SGA vs Healthy AGA, the adj OR is 1.41, p=0.2. Here, the ORs are quite similar, but only for the FGR subgroup is it statistically "significant", since the sample size is bigger (250 FGR, 126 Healthy SGA). The adj OR for AGA with placental dysfunction is 0.93, which is quite close to 1, so for this outcome the data appear consistent with there being an association with SGA, but not placental dysfunction.

On the other hand, for writing at age 7, the adj ORs are 1.46, 0.98, and 1.25 for FGR, AGA with placental dysfunction, and Healthy SGA, respectively. This values are consistent with there being an association with SGA, which is amplified in the presence of placental dysfunction. Without SGA, placental dysfunction has no association with achievement, but in the presence of SGA, placental dysfunction makes achievement in writing at age 7 worse.

A better modelling approach might have been to first assess whether SGA and/or placental dysfunction were independently associated with educational outcomes, and then to test whether there is an interaction. If the FGR group have the lowest educational achievement, this could simply be because they have both risk factors, which independently predict poorer performance. Alternatively, it could be that the FGR group does particularly poorly, which would be evidenced by an interaction between the two exposures.

Unfortunately, there may be little power to detect interactions with this sample size.

**We appreciate the reasoning behind the reviewer’s arguments and, were this a data driven analysis based primarily on the associations observed in our study, we would agree with the approach that we should stratify analyses based on statistical evidence supporting interactions. However, the critical point is that, clinically, differentiating between SGA infants which do – or do not – have indicators of fetal growth restriction is a key element of clinical obstetric practice. Based on the prior clinical knowledge that we have, the stratification of SGA fetuses was planned in advance and was not a result of data driven, post-hoc classification. We have edited the text to emphasise this point (Lines 167-168).**

**With regard to the second point about whether there may be an impact of healthy SGA on educational attainment that we failed to detect based on the point estimates of the OR, we have revised our text to emphasise that the 95% CI for healthy SGA were quite wide for some of the outcomes and that the current analysis should be interpreted in the light of the remaining uncertainty (Lines 249-251).**

Nevertheless, the authors need to avoid simply interpreting their results in terms of whether the p-values are less than 0.05. At least as much emphasis should be put on the sizes of the associations when interpreting the results.

**Thank you; we agree entirely that results should never be interpreted simply in light of which side of an arbitrary cut-off for alpha they happen to fall. We have reported point estimates and 95%CI throughout, and have added additional interpretation where our results indicate an effect, but fall short of the specified p value, i.e. adjusted OR of falling educational standard at age 5 of FGR vs Healthy AGA is 1.33 (0.93-1.89), p=0.11 (Lines 235-236).**

Minor points:

The STROBE checklist is used, but should REPORT extension have been used, since routine data linkage has been performed?

**We have now completed the extension of the existing STROBE guidelines or REporting of studies Conducted using Observational Routinely-collected Data (RECORD) checklist as reporting guidelines for studies conducted using routinely-collected data**

**(S3 Appendix & Line 149-151).**

Line 177 states that "…childhood morbidities not linked to intrauterine development could potentially confound the relationship between perinatal factors and mid-childhood educational attainment." I am probably misunderstand something, but if they are not linked to IU development, how can they be confounders? Besides, why does it matter if the association between perinatal factors and educational achievement is confounded, if that is not the focus of the analysis?

**We apologise for this confusion and have replaced the sentence to: “To minimise the impact of significant childhood morbidities that were not linked to intrauterine development on the analyses, we adjusted for relevant morbidities” (Lines 187-188).**

Reviewer #2: PLOS One
Editor in Chief
December 4, 2022

Re: Laurentya Olga et al. Association between antenatal diagnosis of late fetal growth restriction and educational outcomes in mid-childhood


Comments for the authors:

The study entitled 'Association between antenatal diagnosis of late fetal growth restriction and educational outcomes in mid-childhood' focuses on reading, writing and mathematical skills of children born with fetal growth restriction (FGR). From a clinical point of view, the study is very relevant. FGR studies with long-term outcomes are needed to optimize the timing of the delivery, to counsel the parents and to develop preventive strategies in order to help these children to reach their full academic potential.

**We are in full agreement with the reviewer about the clinical relevance of the study and real-world importance of this data.**

In this cohort study, antenatal assessments included ultrasound examination (estimated fetal weight (EFW), uterine and umbilical artery Doppler measurements) as well as maternal serum concentrations of PAPP-A, AFP, s-Flt and PlGF at 20, 28 and 36 gestational weeks. The study cohort was divided to four antenatal phenotype groups for comparisons of educational outcomes. My comments concerning the manuscript are listed below, the major ones being numbers 2 and 4. I understood that a statistician will separately review the manuscript, and therefore this review does not include any comments on the applied statistics. 


1.      According to CDC (Centers for Disease Control and Prevention) middle childhood is determined as age 6-8 years. Is it appropriate to use term mid-childhood in the title as the study results range from 5-7 years?

**Thank you; the reviewer has prompted us to carefully consider the appropriateness of this term. We appreciate the point that our age range is offset from the CDC definition by 1 year, however we prefer to retain the use of the term as we believe that mid-childhood should be understood by most readers to be around this age range. We have now specified our study age range at the first use of the term ‘mid-childhood’ in the abstract to avoid confusion. The use of 5-7 years age range is ideal to reflect the first 3 years of UK education system where age 5 represents early years/reception evaluation (end of Year 0 of primary school), while age 6 and 7 represent years 1 & 2 assessments, respectively (**[**https://www.gov.uk/national-curriculum**](https://www.gov.uk/national-curriculum) **and S2 Appendix). Given the consistency of the effect sizes we show across the 5-7 year age range, the clinical/developmental implications of the offset from the CDC definition are unlikely to be significant.**

2.      The researchers divided the cohort to four groups antenatally 1) FGR, 2) healthy SGA, 3) appropriate for gestational age (AGA) with markers of placental dysfunction and 4) healthy AGA (reference group).  FGR was defined as EFW < 10th centile and any of the following:  (a) lowest decile of abdominal circumference (AC) growth velocity between 20 and 36 weeks, (b) EFW <3rd centile at 36 weeks, c) umbilical artery pulsatility index in the highest decile at 36 weeks, d) uterine artery pulsatility index in the highest decile at 20 weeks, e) maternal PAPP-A <0.4 MoM at 12 weeks, f) maternal sFlt1:PlGF- ratio >38 at 36 weeks, g) maternal AFP >2.0 MoM at 20 weeks. The researchers explain that they have tried to phenotype the groups carefully in order to distinguish constitutionally small children from children diagnosed antenatally with FGR/placental insufficiency.

My main concern regarding this study is that this phenotyping is not optimal for late FGR. Several FGR inclusion criteria in this study are used to predict the development of FGR/indicate an increased FGR risk, but they are not indicative of FGR per se. The researchers could phenotype FGR in this large cohort more precisely according to internationally accepted criteria for FGR.

**We are slightly surprised at the view that a weakness of the analysis is sub-optimal phenotyping of FGR. The POPS cohort does not – to our knowledge – have a parallel in terms of phenotyping FGR, combining serial blinded ultrasonic fetal biometry, utero-placental Doppler flow velocimetry and biomarkers. The exceptional quality of the phenotyping is manifested in previous publications arising from the cohort, including major papers in the Lancet, Nature, Nature Genetics and Nature Medicine, all of which employ elements of the phenotyping used in the present analysis. It should also be appreciated that fetal growth restriction lacks a gold standard and any definition can be criticised: we have discussed this in detail elsewhere.^1^**

**In relation to the reviewer’s comment, none of the markers indicate FGR “per se”. FGR is a theoretical concept with no gold standard. All measurements are proxies. The indicators that were pre-specified for the present study (S1 Appendix) are all based on extensive literature which have been specifically validated within the POPS cohort. In relation to the comment about internationally accepted criteria, the purely ultrasonic classification is almost identical to the Gordjin 2016 Delphi consensus statement^2^, with the exception that we do not include MCA Doppler as it was not performed in the study. We have expanded the discussion of this point (Lines 311-314).**

--

**^1^Smith GCS. Developing Novel Tests to Screen for Fetal Growth Restriction. Trends Mol Med. 2021 Aug;27(8):743-752. doi: 10.1016/j.molmed.2021.05.006. Epub 2021 Jun 16. PMID: 34147360.**

**^2^Gordijn SJ, Beune IM, Thilaganathan B, Papageorghiou A, Baschat AA, Baker PN, Silver RM, Wynia K, Ganzevoort W. Consensus definition of fetal growth restriction: a Delphi procedure. Ultrasound Obstet Gynecol. 2016 Sep;48(3):333-9. doi: 10.1002/uog.15884. PMID: 26909664.**

In addition, longitudinal development of placental insufficiency, for example umbilical artery PI findings at earlier time points would ensure the readers that only late FGR cases were included. Naturally, it would be also interesting to know the results in various subgroups, formed according to the measured biomarkers, in this exceptional data set.

**The sub-division of FGR into early and late is itself arbitrary, being based on a point of the continuous scale of gestational age. We understand the reviewer’s point, however, as all births included in the present study were at term it is very unlikely that early-onset FGR cases are included.**

3.      The growth charts used in this study are from 1990. Any newer charts available?

**We appreciate that there are a variety of standards that we could have selected for use in this study, however the conversion of birth weight values to sex and age-adjusted SDS using the British 1990 growth reference is in accordance with the UK (RCPCH) guidelines which suggests the use of the British 1990 growth reference at birth (https://www.rcpch.ac.uk/sites/default/files/Using_the_growth_charts.pdf).**

4.      In healthy AGA group, 21% of the children performed below the standard in overall educational attainment at 5 years of age and 23% performed below the standard in writing skills at 7 years of age.  What is the reason that every fourth - fifth AGA child with no signs of placental insufficiency did not to achieve the standard in these educational tests? The educational standard, other health/educational issues in the AGA group despite extensive confounding factor evaluation, teaching, or what? We should be worried about this proportion; one can even argue that this is a greater problem than 5% additional increase in poor performance in writing skills at 7 years in the FGR group compared to this healthy AGA group.

**We understand the reviewer’s concern about this, however these data reflect the nationally bench-marked standards for educational attainment and the percentage of the UK population that meet them. Table 2, below, shows national attainment data, and demonstrates that the POPS population are slightly better than average, likely reflecting the relatively affluent area of study with well-regarded government-funded schooling. Discussion of UK educational standards overall and how to bench-mark achievement is somewhat outside of the scope of this study.**

**Table 2. Percentages of children failing educational standards ages 5-7 years of POPS population vs national average**

|  | **National data (2016)** | **POPS population (2013-2019)** |
| --- | --- | --- |
| **Age 5/GLD** | **30.7%**^1^ | **20%** |
| **Age 6/Phonics (first attempt)** | **19%**^2^ | **12%** |
| **Age 7-Reading** | **26%**^2^ | **16%** |
| **Age 7-Writing** | **35%**^2^ | **22%** |
| **Age 7-Mathematics** | **27%**^2^ | **17%** |
| **Age 7-Science** | **18%**^2^ | **10%** |

---

1.	**Department for Education. *Early Years Foundation Stage Profile Results in England 2019*.; 2019. https://www.gov.uk/government/statistics/early-years-foundation-stage-profile-results-2017-to-2018. Last accessed 17^th^ Feb 2023.**

**2.	Department for Education. *Phonics Screening Check and Key Stage 1 Assessments in England, 2016*. Vol 42.; 2016. Last accessed 17^th^ Feb 2023.**

5.      Could a more detailed discussion on possible mechanisms behind the educational problems in FGR included? The authors discuss that early iatrogenic delivery might have an input. On the other hand, one can argue that delaying the delivery might result in non-optimal outcome.

**Thank you for the prompt regarding potential mechanisms. We have now included more discussion of this topic at Lines 270-276.**

6.      There are publications focusing on reading, writing and other educational outcomes in FGR children with placental insufficiency for example by Geva et al and Partanen et al, which would enhance the findings of the current study and could be discussed. 

**We have now added some discussion of both publications as suggested (Lines 276-278).**

Reviewer #3: The objective of this study was to determine if fetal growth restriction diagnosed in the prenatal period is associated with poorer education in childhood.

The study is based on the pregnancy outcomes prediction study that included unselected nulliparous women between 2008 and 2012. The samples included 4200 patients. Three visits were conducted for research purpose at 20, 28, 36 weeks. Several analytes were measured in peripheral blood including PAPP-A, AFP, PlGF and s-Flt. This study is unique in many ways and could lead to important publication. The outcomes of children in term of education were obtained from the national database in England. Standardized assessments are available for the age of 5, 6, and 7. The article follows the STROKE guidelines which are in Appendix 3. To avoid the confounding effect of preterm birth, the analysis was restricted to infants born at 36 weeks and beyond.

Imputation for covariant was performed using chained equation under a missing at random assumption (detail on page 9).

Results:
*       65% of patients were included in the analysis
*       Mean gestational age at delivery 40 weeks
*       The prevalence of fetal growth restriction was 9%
*       Education at ages 5 and 6: antenatal diagnosis of FGR had a higher rate of not meeting educational standards compared to AGA (lines 220-224).
*       Education at age 7: overall the proportion failing to attend expected education were 16% for reading, 22% for writing, 17% for mathematics and 10% for science. However, the risk was higher for FGR although OR were 1.46-1.49 for the different domains.

Assessment: I find the article very well written and is straightforward. The results are clear, and the conclusions are justified by the data. The results have a lot of information for patients counselling and management.

Questions for the authors:
Fetal growth restriction was defined as a combination of biometric parameters and one or more markers of placental dysfunction. Most obstetrical units use only biometry as an estimated fetal weight less than 10th percentile. What would be the results if the authors use only fetal biometry such as estimated fetal weight below 10th centile, change in abdominal circumference, etc.? I wonder if babies who are SGA without evidence of growth restriction would also have normal education retainment. It would be worth for the obstetrical community to have this information because many around the world do not use PAPP-A or the sFlt-1/PlGF ratio.

**We agree with the reviewer that our study provides important new information in the context of clinical decision-making.**

**We have provided the associations between educational attainment at ages 5-7, fetal growth status, and markers of placental dysfunction separated into scan and biochemical markers separately for this reason (Table A-F in S5 Table). Individual markers are not included due to power considerations. As discussed in Lines 251-253:**

**“The associations were very similar when cases of FGR defined using ultrasonic markers were compared to cases defined by biochemical markers of placental dysfunction.”**
