# Supplementary material for: Association between antenatal diagnosis of late fetal growth restriction and educational outcomes in mid-childhood: A UK prospective cohort study with long-term data linkage study
Source: PLoS Med. 2023 Apr 24;20(4):e1004225. doi: 10.1371/journal.pmed.1004225 (PMC10166482; doi:10.1371/journal.pmed.1004225)
Supplement: Attachment — Submitted filename: Laurentya_Response to reviewers_v2.docx [file pmed.1004225.s013.docx]

26/03/2023

Dear Dr Davidson,

Thank you very much for provisionally accepting our study for publication in *PLOS Medicine*.

We have given careful consideration to all the remaining issues and have addressed these in this resubmission.

Best wishes,

Prof Gordon C S Smith
Head of Department of Obstetrics & Gynaecology, University of Cambridge

Dr Catherine Aiken

Associate Professor and Honorary Consultant in Maternal and Fetal Medicine

University of Cambridge, UK

Requests from Editors:

Please include the study setting in the title.

**We have now included the study setting in the title.**

The Data Availability Statement (DAS) requires revision. For the raw data from the POPS study, please include an appropriate contact (web or email address) for inquiries (this cannot be a study author).

**We have now revised the DAS accordingly.**

Please quantify the key findings from the study in your Author Summary. 

**We have now quantified the key findings from the study in the Author Summary.**

Thanks for including a completed RECORD checklist - when completing the checklist, please use section and paragraph numbers, rather than page numbers (as these will change during the publication process).

**We have now revised the RECORD checklist by indicating sections and paragraph numbers throughout.**

Please remove the Data Availability Statement from the main text and ensure all the relevant information is captured in the submission form questionnaire (your response will be published alongside the article).

**The DAS has now been removed from the main text.**

Changes from the planned analysis should be identified as such in the Methods section of the paper, with rationale.

**We have now identified this in the Methods (Statistical Analyses section, Paragraph 1).**

Comments from Reviewers:

Reviewer #1: Alex McConnachie, Statistical Review

I thank the authors for their consideration of my original points. I am generally happy with their responses.

**Thank you. We appreciate the points raised by Professor McConnachie and are grateful for his input into improving the clarity of our work.**

I note that a power calculation has been added, but this is not reproducible as it stands. It would help if the authors give the assumed percentages in the FGR and healthy AGA groups. If the calculation was done prior to the study, then the numbers required in each group to achieve 80% power should be given. If this calculation was post-hoc, based on the numbers achieved, then this should be stated.

**We have now added this information as requested.**

For the healthy SGA vs. healthy AGA comparisons, it is OK to say that that the CIs are wide, but given the newly added power calculation, it would seem that this comparison is very underpowered. Someone only reading the abstract, lines 44-45, or lines 69-70 in particular, will not get that nuance, and will infer that the only subgroup at increased risk of poor achievement is the FGR group. When making headline statements, the authors could be a little more careful.

**Thank you, we appreciate this point. We have clarified in the abstract that the healthy SGA comparison is under-powered, and removed reference to this comparison from the author summary altogether.**

As an afterthought, I wonder whether there is any possibility that some of those in the healthy SGA group could in fact be undiagnosed FGR - could there be a subgroup of false negatives? What about false positives? How good are the tests for FGR? How would misdiagnoses affect the results? This could, perhaps, be discussed in the limitations section.

**We have now included this in the study limitations. However one of the key characteristics of the POPS study is the robust FGR phenotyping, which does not – to our knowledge – have a parallel, combining serial blinded ultrasonic fetal biometry, utero-placental Doppler flow velocimetry, and biomarkers. The exceptional quality of the phenotyping is manifested in previous publications arising from the cohort, including major papers in the Lancet, Nature, Nature Genetics and Nature Medicine, all of which employ elements of the phenotyping used in the present analysis. It should also be appreciated that fetal growth restriction lacks a gold standard and any definition can be criticised: we have discussed this in detail elsewhere^1^.**

__________________________________________________

1.	Smith GCS. Developing Novel Tests to Screen for Fetal Growth Restriction. *Trends Mol Med*. 2021;27(8):743-752. doi:10.1016/j.molmed.2021.05.006
